# Road Performance of Solid Waste-Based Cementitious Material-Stabilized Reclaimed Base Course Material

**DOI:** 10.3390/ma19071462

**Published:** 2026-04-05

**Authors:** Qi Ma, Jiuguang Geng, Peng Wei, Xijuan Xu, Zewen He, Zhen Wang, Hui Lan

**Affiliations:** 1 School of Materials Science and Engineering, Chang’an University, Xi’an 710064, China; macye0707@163.com (Q.M.); weipeng@chd.edu.cn (P.W.); hezewen@chd.edu.cn (Z.H.); 2 Xi’an Highway Research Institute Co., Ltd., Xi’an 710065, China; h19306308292@163.com; 3 Xi’an Yinding Technology Co., Ltd., Xi’an 710199, China; wangzhen202602@163.com (Z.W.); 13571892032@163.com (H.L.)

**Keywords:** solid waste utilization, reclaimed material, carbon emission, road performance, stabilization

## Abstract

Large-scale pavement maintenance generates substantial amounts of reclaimed base course material (RBM), whose high-value reuse presents a critical challenge. Although cement is commonly used for stabilization, its high carbon footprint and shrinkage issues limit sustainability. This study proposes a 100% solid waste-based cementitious material (SWC) as an alternative stabilizer for pavement base layers containing high proportions of RBM. A comparative investigation was conducted between SWC-stabilized RBM (SSRBM) and ordinary Portland cement-stabilized RBM (CSRBM) regarding key road performance indicators. The results indicate that with 100% RBM, the 7-day compressive strength of SSRBM containing 4% SWC reaches 1.88 MPa, meeting the Chinese specification JTG/T 5521-2019. By incorporating 15% natural coarse aggregate, this strength can be increased by 35.4%. Furthermore, SSRBM demonstrates superior freeze–thaw resistance, with a freeze–thaw-retained unconfined compressive strength ratio of 93.9%, compared to 89.6% for CSRBM, and exhibits a lower drying shrinkage coefficient. Carbon emission analysis shows that the emissions per cubic meter of SSRBM are approximately 73% lower than those of CSRBM, presenting a viable and environmentally advantageous alternative for sustainable pavement construction.

## 1. Introduction

The continuous global expansion of road networks has led to a yearly increase in pavement maintenance and rehabilitation projects, generating substantial quantities of solid waste. In China, over 2.2 million tons of waste pavement materials are produced annually through the reconstruction and rehabilitation of existing highways [1], making reclaimed base course material (RBM) a significant contributor to this waste stream [2]. When large quantities of RBM are left exposed or directly landfilled, they pose substantial risks of resource wastage and environmental pollution [3]. Recent studies have highlighted that the weak mortar attached to recycled aggregate surfaces can significantly influence binder–aggregate affinity and the long-term performance of stabilized materials [4]. Compounding this issue, the rising cost and scarcity of natural aggregates are adversely affecting road construction projects [5]. Therefore, the utilization of RBM in road construction not only addresses disposal challenges but also helps meet the growing demand for raw materials in highway engineering, thereby contributing to environmental protection and sustainable development in the road industry [6,7,8].

Significant progress has been made in the mechanical enhancement, durability assessment, and processing techniques of reclaimed materials for pavement applications. Research underscores the importance of reusing recycled aggregates, requiring detailed characterization and multi-dimensional studies based on quality parameters [9]. Research indicates that partially replacing natural aggregates with recycled ones combines their advantages while mitigating limitations [10]. Carbonation enhances recycled concrete’s compressive strength by 21.05%, though it remains 8.63% lower than natural aggregate concrete [11]. Other studies confirm the viability of using 100% coarse recycled aggregate with low cement content in roller-compacted concrete for low-traffic pavements [12]. Gradation optimization has improved the mechanical and pavement performance of cement-stabilized cold recycled mixtures [13]. Furthermore, the mechanical behavior of construction and demolition waste is strongly influenced by mix composition and compaction conditions, with ceramic particles being particularly prone to breakage and increasing deformability [14]. Using emulsified asphalt with reactive fly ash yields higher early indirect tensile strength, with stiffness and moisture resistance comparable to mixes containing both emulsified asphalt and cement [15]. CO_2_ carbonation both strengthens recycled aggregates and reduces environmental risks in unbound applications [16]. A predictive fatigue model for cement-stabilized bases has been established through systematic testing [2], and an optimal recycled brick masonry incorporation rate of 20% has been identified, with fiber reinforcement significantly improving strength and deformation resistance [1].

Nevertheless, a common thread in these studies is that increasing the recycled material content generally leads to a deterioration in performance, necessitating additional treatments or higher cement dosages [10,11,17]. These approaches often compromise the environmental benefits of recycling. The key challenges stem from the inherently weak interfacial transition zone (ITZ) of RBM, its variable gradation, and the increased shrinkage potential at higher binder contents. Moreover, the continued reliance on ordinary Portland cement as the primary stabilizer constrains the potential for truly low-carbon road construction. An integrated structural–environmental LCA study by Santos et al. on unbound and cement-stabilized recycled aggregates for subbase layers confirmed that while cement stabilization reduces the required pavement thickness, its environmental benefits are outweighed by the impact associated with cement usage, further supporting the need for alternative and more environmentally friendly binders [18].

While multi-component solid waste-based cementitious materials have been extensively studied in Western countries where their application is relatively mature, their widespread adoption in China still faces context-specific challenges. First, industrial by-products such as steel slag and fly ash exhibit significant compositional variations across Chinese provinces due to differences in local industries and raw material sources [16]. Studies have shown that solid waste treatment efficiency varies considerably across regions, with western China achieving 0.65 compared to 0.53 in the eastern region [19], further reflecting the regional disparities in waste characteristics and management practices. Second, while laboratory studies have demonstrated the technical feasibility of such materials, long-term field performance validation under China’s diverse climatic and traffic conditions remains limited [20]. The gap between laboratory research and practical application constrains widespread adoption. These region-specific challenges underscore the need for continued research tailored to the Chinese context.

The increasing urgency of mitigating climate change and the persistent challenge of shrinkage-induced cracking in cement-stabilized materials provide strong motivation for developing alternative binders that are both low-carbon and crack-resistant. To address these challenges, this study proposes an alternative approach for the direct utilization of high-content RBM stabilized with a solid waste-based cementitious material (SWC). The proposed SWC is synthesized entirely from industrial by-products, representing a 100% waste-based binder. Unlike conventional methods, the proposed strategy avoids energy-intensive treatments such as carbonation curing, high-temperature processing, or complex chemical additives, thereby significantly reducing carbon emissions and energy consumption. The mechanical properties, durability performance, and environmental impacts of SWC-stabilized RBM are systematically evaluated and compared with those of conventional cement-stabilized mixtures. Through laboratory testing and microstructural analysis, this research aims to demonstrate the feasibility of using SWC-stabilized high-content RBM as a sustainable alternative for pavement base construction, thereby promoting large-scale and low-carbon recycling of reclaimed road materials [21].

## 2. Raw Materials and Test Methods

### 2.1. Raw Materials

#### 2.1.1. Aggregates

**Figure 1:**
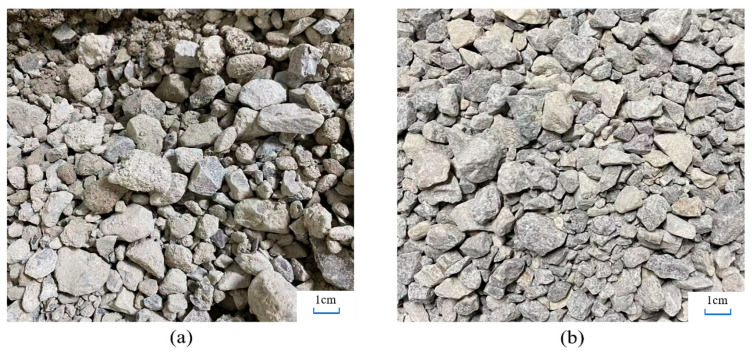
Aggregate appearance (**a**) RBM; (**b**) NA.

The reclaimed base course material (RBM) used in this study was sourced from Dongtao Road, Tongchuan City, China. Initial gradation analysis of the RBM (Table 1) revealed a maximum nominal particle size of 31.5 mm with no missing size fractions, indicating a well-graded particle size distribution. The gradation analysis was performed in accordance with the Chinese standard JTG 3432-2024 “Test Methods of Aggregate for Highway Engineering” [22]. The new materials consist of two grades of limestone natural aggregate (NA): the first grade is 0–10 mm, abbreviated as NA-F, and the second grade is 10–20 mm, abbreviated as NA-C. Both grades were sourced from a quarry in Xi’an. The appearance of both aggregates is shown in Figure 1. The RBM exhibits a surface covered with cement mortar, resulting in a rough and porous texture, while the natural aggregate surface remains angular with a smooth surface and only minor stone powder residue.

**Figure 2:**
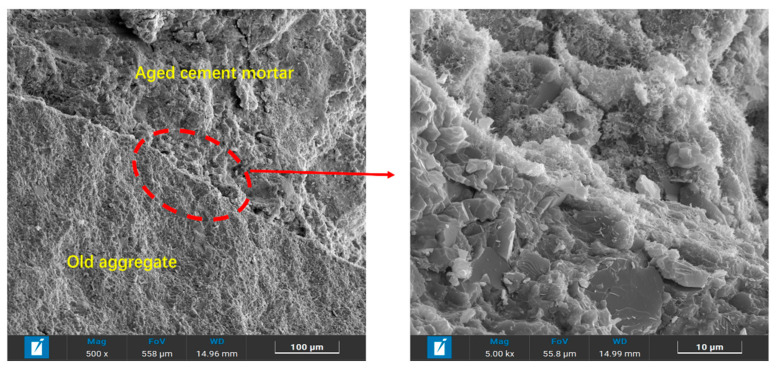
Microstructural features of RBM.

Scanning electron microscopy (SEM, TESCAN CLARA, Brno, Czech Republic) was used to examine the microstructure of the interface transition zone (ITZ) in the RBM. Samples were gold-coated prior to observation to ensure conductivity. The images, taken at 500× and 5000× magnification under standard operating conditions, are shown in Figure 2. The upper portion consists of old OPC mortar, while the lower portion comprises old aggregate. Magnified ITZ images reveal an irregular and uneven surface on the old aggregate. The overlying old cement mortar primarily contains AFT, C-S-H gel, and CaCO_3_ products, with microcracks appearing at their contact interface.

Table 2 and Table 3 indicate that the RBM exhibits higher water absorption, crushing value, needle sheet content, and soft stone content compared to NA. The physical properties of both coarse and fine aggregates presented in Table 2 and Table 3 were determined according to the Chinese standard JTG 3432-2024 “Test Methods of Aggregate for Highway Engineering”. This demonstrates that after long-term service and milling damage, the RBM deteriorates in compactability, mechanical strength, and angularity. This phenomenon is corroborated by the aggregate’s apparent morphology observed in Figure 2. Notably, the milling process generates microcracks within the aggregate, which significantly contributes to the decline in strength and angularity [5]. However, when the RBM is reused, the newly added SWC slurry and cement slurry can fill voids and enhance interfacial bonding, potentially mitigating these issues.

#### 2.1.2. Cementitious Materials

The cement used in the test was P.O42.5 ordinary Portland cement (OPC) produced by Conch Cement Factory (Wuhu, China). The solid waste cementitious material employed was developed earlier by the research team and primarily comprised industrial solid wastes including steel slag, desulfurization gypsum, blast furnace slag, red mud, and fly ash.

**Figure 3:**
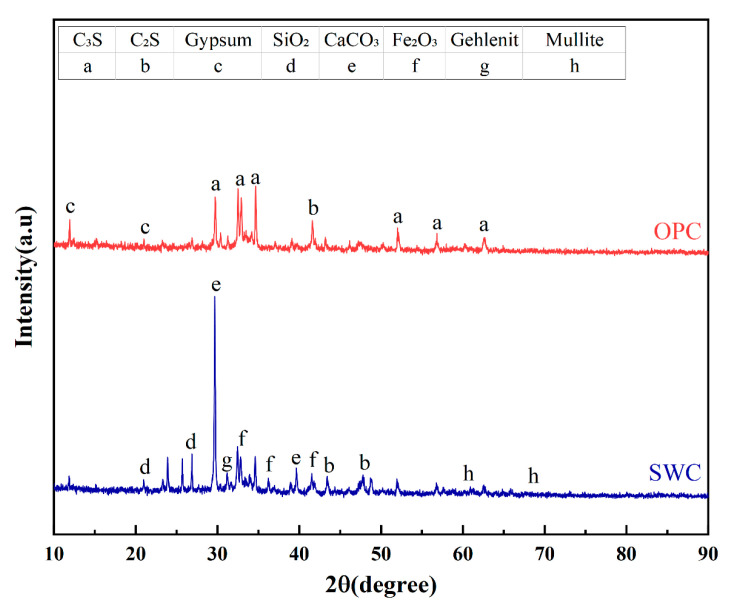
XRD of OPC and SWC powder.

The X-ray fluorescence (XRF) results (Table 4) and X-ray diffraction (XRD) analysis results (Figure 3) for OPC and SWC powder are shown below. The XRF analysis was conducted using a PANalytical Axios wavelength dispersive X-ray fluorescence spectrometer (Malvern Panalytical, Almelo, The Netherlands) equipped with a rhodium (Rh) target X-ray tube operating at up to 60 kV and 160 mA, with a maximum power of 4 kW. According to XRF testing, the primary chemical compositions of OPC and SWC showed no significant differences. The Ca/Si ratio was 2.21 for OPC and 2.26 for SWC. The XRD analysis was performed using a PANalytical Empyrean diffractometer with Cu Kα radiation, operating at 45 kV and 40 mA, with a scanning range of 5° to 90° 2θ. The diffraction peaks of OPC are primarily composed of tricalcium silicate (3CaO·SiO_2_), dicalcium silicate (2CaO·SiO_2_), and gypsum (CaSO_4_·2H_2_O) [23]. In contrast, the SWC diffraction pattern exhibits distinct differences, with its peaks primarily composed of quartz, CaCO_3_, Fe_2_O_3_, gehlenite, and mullite [24]. 

The physical and mechanical performance indicators of both cementitious materials presented in Table 5 were tested in accordance with the Chinese standard JTG 3420-2020 [25] “Testing Methods of Cement and Concrete for Highway Engineering”. Table 5 reflects the fundamental performance indicators of SWC and OPC. It is evident that SWC exhibits a larger specific surface area and higher standard consistency water requirement than cement, indicating that SWC requires more water than OPC. These characteristics are consistent with those of multi-component blended cementitious systems reported in the literature, where the combination of industrial by-products often results in increased water demand due to higher fineness and particle morphology [26]. Additionally, the compressive and flexural strength test results for SWC are lower than those for cement.

**Figure 4:**
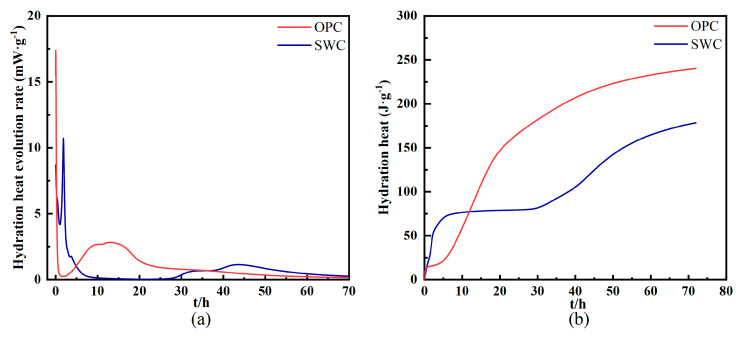
Hydration of OPC and SWC: (**a**) hydration rate and (**b**) hydration heat.

The hydration heat release rate and total heat of hydration test results for both cementitious materials are shown in Figure 4. The measurements were conducted using an isothermal calorimeter (The I-Cal 8000 HPC Calorimeter, Calmetrix Inc., Needham, MA, USA) at 20 °C for 72 h. SWC exhibits a significantly higher hydration heat release rate than OPC within the 0–5 h period, with a peak at 1.86 h, while OPC peaks at 0.03 h. The total heat of hydration after 72 h is 478,133 J/g for SWC and 734,751 J/g for OPC. Although SWC exhibits a higher hydration reaction rate than OPC during the first 5 h, OPC subsequently catches up and even surpasses it.

#### 2.1.3. Gradation Design

**Figure 5:**
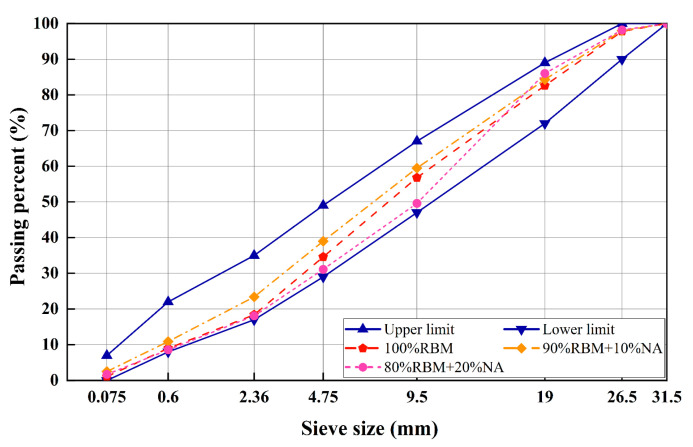
Gradation curve.

For the recycling of water-stabilized old materials, China’s Technical Specification for Recycling of Highway Asphalt Pavements (JTG/T 5521-2019) [27] provides relevant regulations. The gradation range selected follows the recommended range in JTG/T 5521-2019, with the gradation curve shown in Figure 5. This study designed 3 gradations and 7 cementitious material dosages, totaling 21 groups. The three gradations are: 100% RBM, 90% RBM + 10% NA-F, and 80% RBM + 5% NA-F + 15% NA-C. This is based on the literature review and preliminary experiments conducted earlier [6,10,16,17,23,28]. The seven cementitious material dosages are: 4% SWC, 5% SWC, 6% SWC, 7% SWC, 3% OPC, 4% OPC, and 5% OPC.

For ease of identification in the subsequent analysis, each mixture is designated by a code. The first number indicates the RBM content percentage (e.g., 100, 90, 80). The following numeral indicates the type and dosage of stabilizer: Arabic numerals (4, 5, 6, 7) represent SWC dosages of 4%, 5%, 6%, and 7%, respectively; Roman numerals (III, IV, V) represent OPC dosages of 3%, 4%, and 5%, respectively. For example, “100-4” denotes the mixture with 100% RBM and 4% SWC, while “80-III” denotes the mixture with 80% RBM and 3% OPC.

### 2.2. Test Methods

#### 2.2.1. Compaction Test

The compaction test was conducted according to the Chinese standard JTG 3441-2024 using the modified Proctor method. The mixture was compacted in a φ152 mm × 170 mm mold using a 4.5 kg rammer with a hammer face diameter of 5 cm, and the average unit compaction energy was 2.687 J. After drying the sample, the moisture content and dry density of the mixture were determined to calculate its maximum dry density and optimum moisture content for forming specimens. Since the compaction energy remains constant for each test, compacting the base course mixture at different moisture contents yields varying dry densities. As moisture content increases, the dry density after compaction gradually rises. Beyond a certain moisture content range, further increases cause the dry density to decrease.

#### 2.2.2. Unconfined Compressive Strength Test

Cylindrical specimens (150 mm diameter, 150 mm height) were prepared at 98% compaction using the optimum moisture content and maximum dry density. After molding, specimens were cured in a standard curing chamber (temperature 20 °C, relative humidity 98%) until specified ages (7, 28, 60, 90 days). Unconfined compressive strength (UCS) was conducted per the “Test Procedures for Inorganic-Bonded Stabilized Materials in Highway Engineering” (JTG 3441-2024), with strength values recorded. One day prior to testing, specimens were immersed in water for 24 h. The loading rate was 1 mm/min.

#### 2.2.3. Indirect Tensile Strength Test

Cylindrical specimens (150 mm diameter, 150 mm height) were prepared at 98% compaction using the optimum moisture content and maximum dry density. After molding, specimens were cured in a standard curing chamber (temperature 20 °C, relative humidity 98%) for specified durations (7, 28, 60, 90 days). The splitting tensile strength (ITS) test was conducted per the “Test Procedures for Inorganic-Bonded Stabilized Materials in Highway Engineering” (JTG 3441-2024), with strength values recorded. One day prior to testing, specimens were immersed in water for 24 h. The loading rate was 1 mm/min.

#### 2.2.4. Flexural Tensile Strength Test

Mid-beam specimens with dimensions of 100 mm × 100 mm × 400 mm were prepared at 98% compaction using the optimum moisture content and maximum dry density. After molding, the specimens were cured in a standard curing chamber (temperature 20 °C, relative humidity 98%) for specified durations (7, 28, 60, and 90 days). The flexural tensile strength test was conducted in accordance with the Chinese standard JTG 3441-2024 “Test Methods of Materials Stabilized with Inorganic Binders for Highway Engineering”, with strength values recorded. The loading rate was controlled at 50 mm/min.

#### 2.2.5. Compression Resilience Modulus Test

Cylindrical specimens with a diameter of 150 mm and a height of 150 mm were prepared at 98% compaction using the optimum moisture content and maximum dry density. After molding, the specimens were cured in a standard curing chamber (temperature 20 °C, relative humidity 98%) for 7, 28, 60, and 90 days. The compression resilience modulus of the specimens was tested using the top surface method in accordance with the Chinese standard JTG 3441-2024 “Test Methods of Materials Stabilized with Inorganic Binders for Highway Engineering”.

#### 2.2.6. Dry Shrinkage Test

Prepare 100 mm × 100 mm × 400 mm beam specimens at 98% compaction under optimum moisture content. After standard curing for 7 days (with the final day involving 24 h immersion in water), conduct the dry shrinkage test. Weigh the specimens at 0, 1, 2, 3, 4, 5, 6, 7, 14, and 28 days after test initiation, recording shrinkage deformation. The test was performed in accordance with the Chinese standard JTG 3441-2024 “Test Methods of Materials Stabilized with Inorganic Binders for Highway Engineering”.

#### 2.2.7. Freeze–Thaw Test

The freeze–thaw resistance was evaluated using 5 cycles, as specified in JTG 3441-2024 for semi-rigid base materials cured for 28 days. This study focuses on comparing the relative frost resistance of SSRBM and CSRBM under identical conditions. Previous research indicates that the most significant deterioration occurs within the first 5–10 cycles for cement-stabilized materials, and that 5-cycle results correlate reasonably well with overall frost resistance rankings [29,30,31]. Therefore, for the purpose of this comparative study, the 5-cycle protocol was considered sufficient to distinguish the relative performance of different stabilizers. It is acknowledged that longer-term cycling (e.g., 25–50 cycles) would provide additional insights into progressive deterioration and is recommended for future research.

#### 2.2.8. Micro-Mechanism Analysis of SSRBM and CSRBM

To investigate the hydration products of SSRBM and CSRBM, this study prepared mixtures with 100% RBM content stabilized with 4% OPC and 4% SWC, respectively. The primary focus lies in evaluating the influence of different stabilizers on the hydration products of the mixtures. The specimens were cured under controlled conditions at 20 ± 2 °C and 98% relative humidity for 28 days. Subsequently, the cured samples were ground and analyzed using X-ray diffraction (XRD). To examine the microstructure of the mixtures, scanning electron microscopy (SEM) was employed to observe the microstructural characteristics of SSRBM and CSRBM after 28 days of curing.

#### 2.2.9. Carbon Emission and Cost Analysis

This study evaluated the CO_2_ emissions and economic aspects of SSRBM and CSRBM based on the material quantities per cubic meter. The carbon emissions were calculated using the unit emission factors, and the material consumption for each mixture is summarized. It should be noted that the optimum moisture content calculations for both mixtures accounted for the inherent moisture present in RBM, NA, SWC, and OPC. Assuming all materials are sourced locally, transportation-related emissions and costs were excluded from the analysis. The following sections present the carbon emission results followed by a preliminary discussion of the economic considerations.

## 3. Results and Discussion

### 3.1. Compaction Properties

The optimum moisture content (OMC) and maximum dry density (MDD) of mixtures significantly influence pavement performance. Table 6 presents the OMC and MDD values for varying gradations and cementitious material dosages. As the dosage of cementitious material increases, both the MDD and OMC generally increase. However, this trend alters when the gradation is modified. With the addition of 10% NA-F, the OMC decreases from 11.5% to 11.2%, while the MDD increases from 2.129 g/cm^3^ to 2.132 g/cm^3^. When a higher proportion of NA-C is incorporated, the OMC further reduces to 10.7%, and the MDD rises to 2.135 g/cm^3^. This behavior can be attributed to the lower water absorption and higher density of natural aggregates (NA), which alter the MDD and OMC relationship when blended with RBM. From the perspective of binders, ordinary Portland cement (OPC) results in a lower OMC but a higher MDD compared to high-silicate cement (SWC). This is primarily due to the larger specific surface area of SWC, which requires more water during compaction, consequently leading to a reduction in MDD.

It is important to note that while the observed increases in MDD (e.g., from 2.129 to 2.139 g/cm^3^) are relatively small, this is a consequence of the dominant role of the RBM aggregate skeleton in compaction. Despite the modest numerical increments, the MDD consistently increases with higher binder content across all gradations, indicating a systematic effect of the cementitious material on the compaction characteristics of the mixtures.

### 3.2. Unconfined Compressive Strength (UCS)

**Figure 6:**
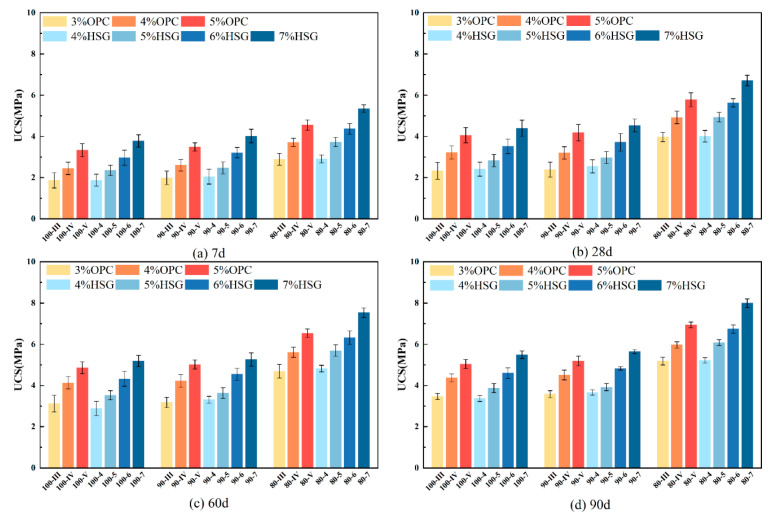
UCS of different age stages and ratios.

Figure 6 shows the 7-day, 28-day, 60-day, and 90-day UCS values and their standard deviations for the 21 mix designs. UCS increases with higher binder content and longer curing periods. At the same binder content, the UCS variation between the 90% and 100% binder groups is negligible, but the 80% binder group exhibits significant variation. This indicates that NA-F contributes minimally to improving mixture strength, while NA-C significantly enhances mixture strength. Increasing the coarse aggregate proportion enhances interlocking and friction forces among mixture particles, leading to a marked increase in compressive strength. At the 7-day age, the 80-4 mixture exhibits a UCS of 2.91 MPa and the 80-III mixture of 2.89 MPa, while the 100-4 mixture recorded 1.88 MPa and the 100-III mixture 1.87 MPa, representing strength increases of 55% and 54%, respectively. Therefore, under identical gradation, the 3% OPC dosage achieves strength equivalent to 4% SWC, with this conclusion applicable to other dosages.

Table 7 outlines the technical requirements [27] for the 7-day unconfined compressive strength (UCS) of cement-stabilized recycled mixture. A comparison between the experimental results and the specification requirements reveals that, for the base layer, only three mix groups—all from the 80% gradation group with high ordinary Portland cement and high-strength mortar content—met the stringent UCS criteria for extreme-heavy and ultra-heavy traffic loads. Nine groups satisfied the design requirements for heavy traffic loads, while fourteen groups complied with the specifications for medium-light traffic conditions. In contrast, the design standards for the subbase layer are less demanding. Twelve mix groups met the specifications for ultra-heavy and special traffic conditions, fifteen groups fulfilled the requirements for heavy traffic loads, and eleven groups aligned with the standards for medium–light traffic. Based on this distribution, CSRBM is more suitable for use in base layers under heavy, medium, and light traffic conditions, while also demonstrating broad applicability in subbase applications.

**Figure 7:**
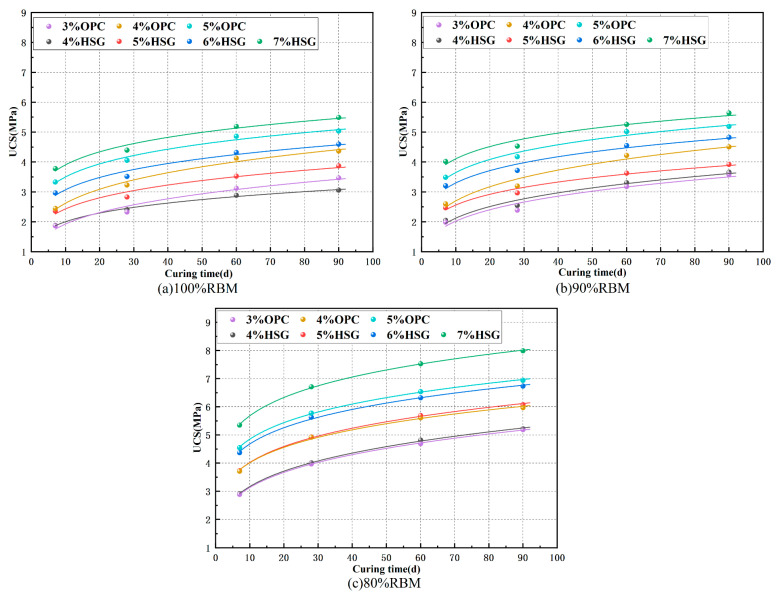
UCS and curing age fitting curve.

To better analyze the relationship between UCS and curing time, the UCS data for the three gradations were fitted against curing time. The results of the power function fitting are shown in Figure 7, with the fitting equation being y = ax^b^. The values of a, b, and R^2^ are presented in Table 8. The fitting indicates a high correlation coefficient R^2^ for the power function curve regression, suggesting excellent fitting results and more accurate strength prediction for subsequent ages. It can be observed that the strengths of the 100% RBM and 90% RBM groups are similar at each mix ratio. The addition of 10% NA-F weakens the interlocking effect between particles in the mixture, contributing little to strength enhancement and potentially reducing shrinkage resistance, thereby increasing cracking risk. Compared to the 90% RBM and 100% RBM groups, the 80% RBM group exhibited a greater strength increase, indicating that adding 15% NA-C significantly enhances mixture strength, further supporting the earlier conclusion.

### 3.3. Indirect Tensile Strength (ITS)

**Figure 8:**
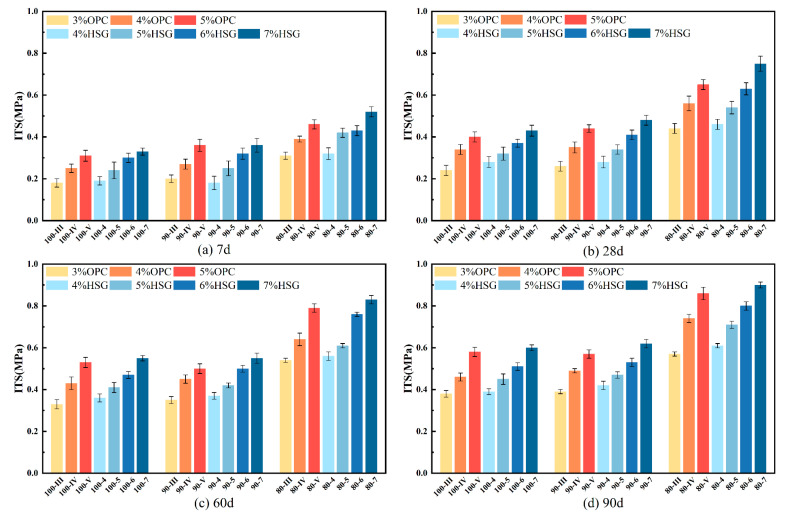
ITS of different age stages and ratios.

ITS results are shown in Figure 8. At 7 days, the minimum ITS value was 100-III, measuring 0.18 MPa, equivalent to 9.6% of the UCS. It is evident that increasing the cementitious material content enhances the formation of hydration products, while extended curing time allows for more complete hydration reactions. This trend aligns with the UCS test results, indicating a positive correlation between the two. Increasing the proportion of cementitious materials enhances bonding performance and improves interfacial structure. The 10% NA-F added to the 90% RBM mixture contributed little to splitting strength growth, while the 15% NA-C added to the 80% RBM mixture significantly influenced splitting strength growth. The 80% RBM group exhibited the best crack resistance. As can be observed from the figure, the ITS values corresponding to cement contents of 3%, 4%, and 5% are comparable to those of SWC contents of 4%, 5%, and 6%, respectively. This indicates that achieving equivalent crack resistance in SSRBM compared to CSRBM requires an approximately 1% increase in stabilizer content.

The observed differences in ITS between SSRBM and CSRBM can be attributed to distinct microstructural developments, as evidenced by the SEM and XRD analyses in Section 4. First, SSRBM forms a fibrous C-S-H network, which effectively bridges micro-cracks and distributes tensile stress, thereby enhancing resistance to splitting failure. In contrast, CSRBM exhibits a clustered C-S-H morphology that is less effective at resisting tensile stress due to stress concentration at cluster boundaries.

Furthermore, XRD analysis reveals that CSRBM generates a higher content of portlandite (Ca(OH)_2_), which tends to accumulate at the interfacial transition zone (ITZ) and form oriented crystals. This Ca(OH)_2_-rich layer is inherently weak and susceptible to micro-cracking under tensile loading, directly compromising ITS. SSRBM, with its lower Ca(OH)_2_ content, avoids this ITZ vulnerability.

Additionally, the fibrous C-S-H network in SSRBM implies a refined pore structure, reducing the number of large capillary pores that act as stress concentrators. These combined microstructural factors, including C-S-H morphology, phase composition, and pore characteristics, collectively explain the superior crack resistance of SSRBM, particularly in the 80% RBM group, where improved gradation further enhances the ITZ.

**Figure 9:**
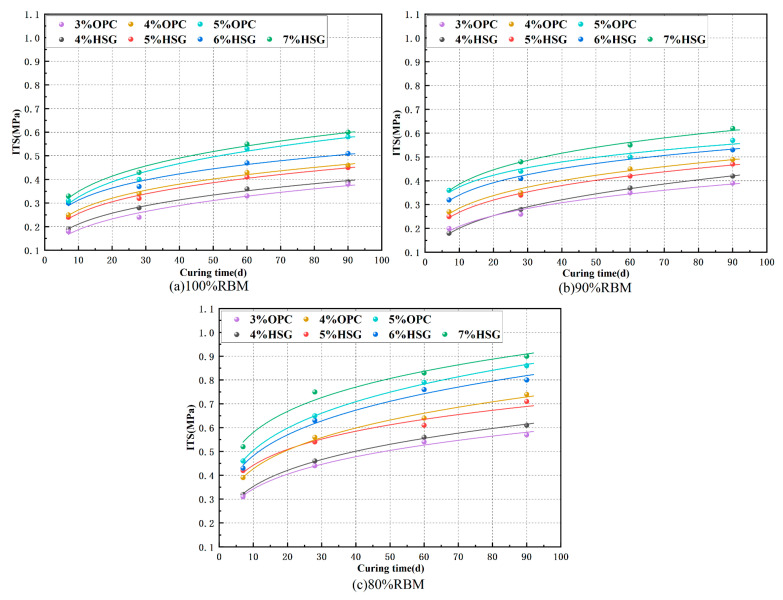
ITS and curing age fitting curve.

Figure 9 shows the fitted relationship between ITS and curing age, with the fitting equation y = ax^b^. The values of a, b, and R^2^ are presented in Table 9. The high R^2^ value indicates a highly reliable fitting relationship. Notably, at an 80% RBM mix ratio, the strength growth curve from 7 to 28 days exhibits steep inclines, indicating vigorous hydration reactions for both OPC and SWC. After 28 days, the growth rate moderates, and the curve gradually flattens.

### 3.4. Flexural Strength

**Figure 10:**
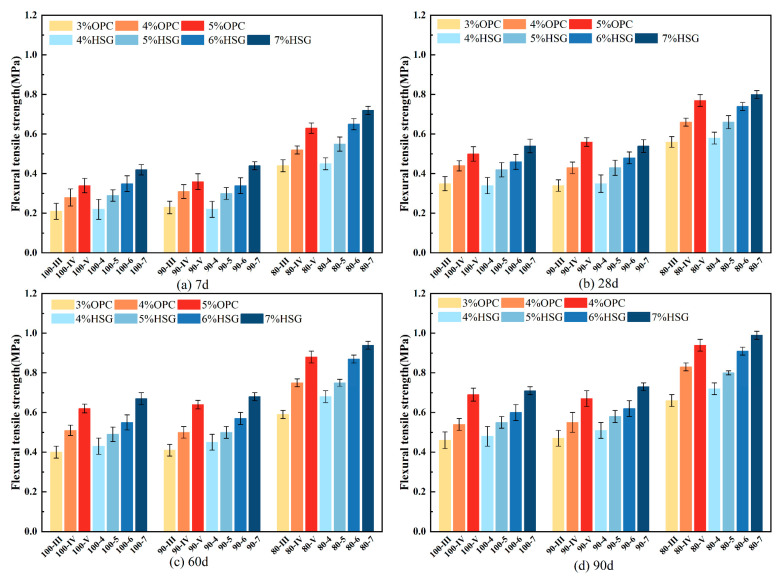
Flexural strength results.

Figure 10 presents the flexural strength test results of various mixtures at different curing ages. Analysis indicates that the flexural strength of the mixtures exhibits a positive correlation with both curing age and stabilizer dosage. The most significant increase in flexural strength occurs when the curing age extends from 7 days to 28 days, suggesting this period is critical for strength development. Beyond 28 days, the rate of strength gain slows considerably, which is consistent with the deceleration of later-stage hydration reactions observed in the hydration heat analysis. It should be noted that while the rate of strength gain decreases after 28 days, hydration reactions continue to a certain extent, contributing to further microstructural densification.

The trend observed for flexural strength is consistent with that of splitting tensile strength, both of which serve as important indicators for evaluating the tensile damage and deformation performance of inorganic binder-stabilized materials. This consistency can be attributed to the fact that both ITS and flexural strength are governed by similar microstructural mechanisms. As discussed in Section 3.3, the formation of a fibrous C-S-H network in SSRBM effectively bridges micro-cracks and distributes tensile stress, which similarly enhances resistance to flexural deformation [32]. In contrast, the clustered C-S-H morphology in CSRBM, combined with higher portlandite content at the interfacial transition zone (ITZ), creates weak regions that are susceptible to cracking under both splitting and flexural loading [33].

At the 90-day curing mark, the flexural strength values for groups 100-III, 90-III, and 80-III are 0.46 MPa, 0.47 MPa, and 0.66 MPa, respectively. The mixture with 90% RBM shows a flexural strength comparable to the 100% RBM group, with no significant difference. In contrast, the 80% RBM group demonstrates a substantial strength improvement, with an increase of approximately 43.5%. This result implies that the addition of 10% NA-F has a negligible effect on flexural strength, whereas the enhancement is primarily attributable to the incorporation of 15% NA-C in the 80% RBM group. The introduction of coarse aggregates optimizes the gradation of the 80% RBM mixture, resulting in a denser structure and enhanced interlocking effects. This finding is consistent with previous studies on recycled aggregate mixtures, which have reported that improved gradation and increased coarse aggregate content enhance the mechanical performance of stabilized materials by improving particle interlock and reducing porosity [34,35]. Furthermore, it increases the contact area and interfacial bonding capacity with the cementitious matrix, thereby improving the mixture’s resistance to flexural deformation during the elastic stage.

Additionally, the flexural strength of group 100-III (with 3% cement content) is 0.46 MPa, which is comparable to the 0.48 MPa value of group 100-4 (with 4% SWC content). This indicates that a 3% cement dosage can achieve a flexural strength level equivalent to that achieved with a 4% SWC dosage. This pattern holds across different curing ages and gradation conditions. As shown in Table 4, the calcium content of SWC is lower than that of cement, suggesting a relatively lower proportion of effective active components in SWC. This observation aligns with studies on multi-component blended cementitious systems, where the dilution effect and slower reaction kinetics of supplementary cementitious materials result in lower early-age strength compared to pure Portland cement [36,37]. Consequently, at an equivalent dosage, the strength performance of SSRBM is inferior to that of CSRBM.

### 3.5. Compression Resilience Modulus

**Figure 11:**
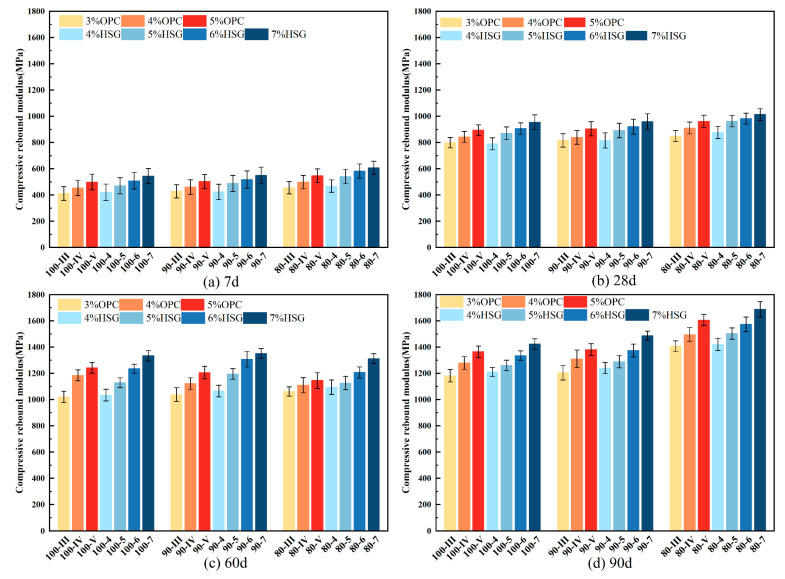
Compression resilience modulus results.

The compressive resilient modulus is a key parameter in pavement structure design, reflecting the material’s ability to resist elastic deformation under vertical loading. This parameter is influenced not only by the bond strength within the interfacial transition zone between aggregates but also closely related to the pore structure, density, and overall compactness of the mixture [2,8]. Figure 11 presents the test results of the compressive resilient modulus for mixtures with varying amounts of RBM at curing ages of 7 d, 28 d, 60 d, and 90 d. It can be observed that as the RBM content decreases, the compressive resilient modulus of the mixtures generally shows an upward trend, a pattern consistent with the unconfined compressive strength (UCS) and the indirect tensile strength (ITS). This positive correlation between resilient modulus and strength parameters has been widely reported in studies on recycled aggregate mixtures [16,17].

Analysis suggests that the hydration products adhering to the surface of RBM result in lower internal friction resistance compared to natural aggregates, which is unfavorable for enhancing the resilient modulus. Similar findings have been reported by Aboutalebi Esfahani [2], who observed that the residual mortar on recycled aggregates significantly affects the mechanical behavior of stabilized mixtures. For instance, while the 90% RBM group incorporated 10% natural fine aggregate (NA-F), the 80% RBM group included 15% natural coarse aggregate (NA-C) along with 5% NA-F. The incorporation of coarse aggregates contributes to the formation of a more stable skeletal structure, while fine aggregates effectively fill the inter-particle voids, thereby improving overall compactness and stiffness. This gradation optimization effect on resilient modulus is consistent with observations by Cai et al. [8] and Yan et al. [35], who demonstrated that improved particle packing enhances the load-bearing capacity of cement-stabilized recycled mixtures. Consequently, the 80% RBM group exhibited the highest compressive resilient modulus. At the 90 d curing age, the resilient modulus of the 80-7 group reached 1689 MPa, compared to 1566 MPa for the 100-7 group, representing an increase of approximately 7.9%. These values are within the typical range reported for cement-stabilized recycled materials [34,38].

This performance improvement is primarily attributed to the inherent high porosity and low strength of RBM. The residual old mortar on the surface of RBM weakens the interfacial bond with the new cementitious matrix, reducing load transfer efficiency, as previously documented in studies on recycled aggregate concrete [28,39]. The appropriate incorporation of natural aggregates reduces the proportion of RBM, enhances the bonding performance at the aggregate–cement paste interface, increases inter-particle friction, and mitigates the influence of weak zones, thereby effectively improving the compressive resilient modulus. In summary, the rational incorporation of natural coarse aggregates into RBM can optimize the mechanical properties of the mixture, particularly in terms of enhancing the compressive resilient modulus, which aligns with the conclusions of recent studies on sustainable pavement materials [11,18].

### 3.6. Dry Shrinkage

**Figure 12:**
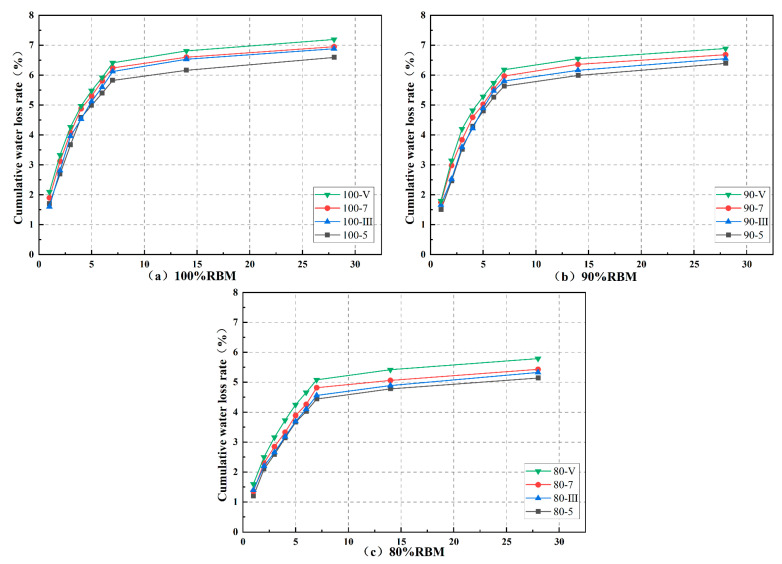
Cumulative water loss rate.

**Figure 13:**
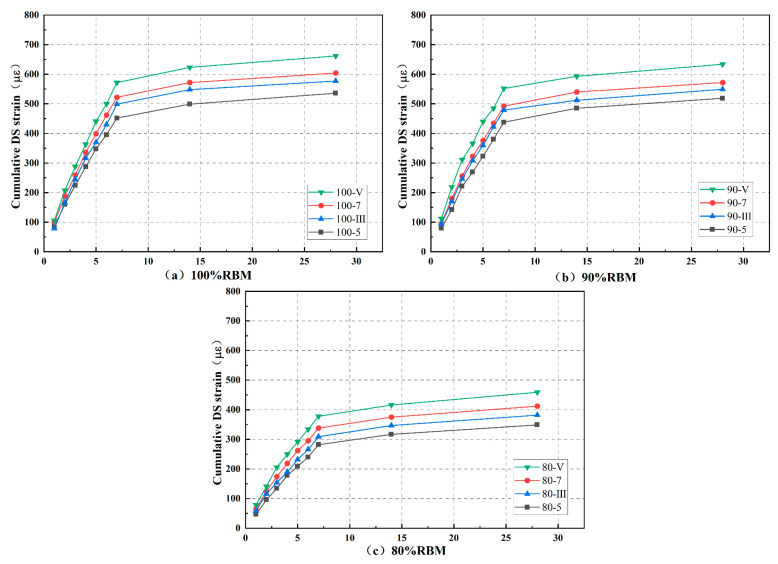
Dry shrinkage strain.

**Figure 14:**
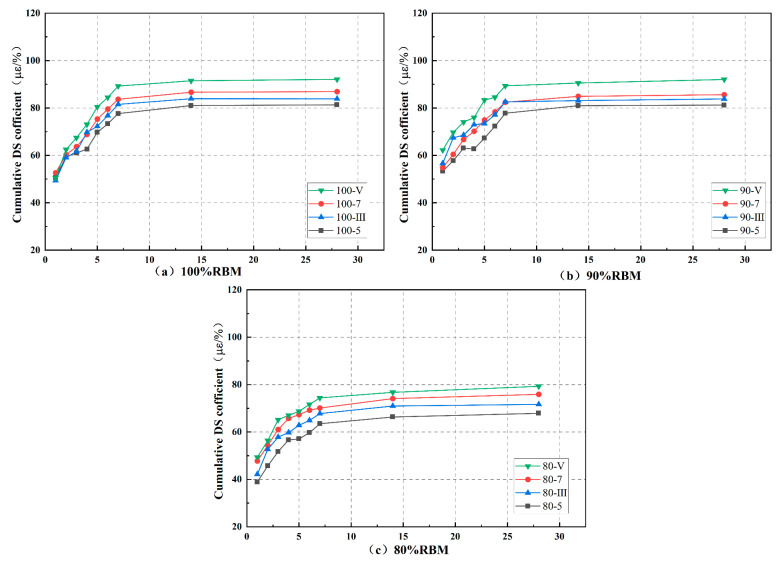
Dry shrinkage coefficient.

Each gradation in the drying shrinkage test had four cementitious material dosages, totaling 12 groups: 100-5, 100-7, 100-III, 100-V, 90-5, 90-7, 90-III, 90-V, 80-5, 80-7, 80-III, and 80-V. The cumulative water loss rate, dry shrinkage strain, and dry shrinkage coefficient are shown in Figure 12, Figure 13 and Figure 14.

The mixture undergoes macroscopic volume shrinkage due to reduced internal moisture content. This shrinkage results from the coupled effects of capillary tension, intermolecular forces between adsorbed water molecules, and interlayer water evaporation [17,28]. During the first 7 days, cumulative water loss rate, dry shrinkage strain, and dry shrinkage coefficient all exhibit an upward trend, leveling off after 7 days. This indicates that early-stage curing should be prioritized when using RBM to prevent cracking. The increased water loss rate in the first 7 days results from the rapid release of free water from the mixture’s pores; while the loss rates of capillary water, adsorbed water, bound water, and interlayer water are extremely slow, gradually reducing the overall water loss rate.

Additionally, increasing the cementitious material content also reduces the mixture’s resistance to drying shrinkage. This is because higher cementitious content increases the mixture’s water demand, leading to larger overall volumes of hydration products. When water evaporates, these products are more prone to shrinkage and cracking. This phenomenon, however, cannot be attributed solely to the increased volume of hydration products; rather, it involves more complex changes in the pore structure. A higher binder content typically leads to pore refinement, where the volume of large capillary pores decreases while the volume of fine mesopores increases. These finer pores generate higher capillary tension during water evaporation, which directly contributes to increased drying shrinkage. Moreover, the figure demonstrates that at identical admixture ratios, CSRBM exhibits inferior dry shrinkage resistance compared to SWC. Previous analyses of hydration kinetics indicate that SWC exhibits lower total hydration heat than OPC, suggesting a lesser overall quantity of hydration products formed. Cement produces a larger volume of hydration products, including C-S-H gel and Ca(OH)_2_. However, as shown in the microstructural analysis, the C-S-H gel formed in SWC has a fundamentally different morphology (a fibrous network) compared to the clustered gel in OPC. This fibrous network, despite arising from a lower total product volume, creates a more effective three-dimensional framework for resisting shrinkage stresses, which explains the superior drying shrinkage resistance of SSRBM. Similar observations on the relationship between C-S-H morphology and shrinkage behavior have been reported in studies on recycled aggregate mixtures [34,35].

By incorporating clean natural aggregates with low water absorption and high shrinkage resistance while reducing the proportion of RBM, both the cumulative water loss rate and dry shrinkage strain of the mixture decrease. However, this is closely related to the type of natural aggregate. The 90% mixture with 10% NA-F showed inferior resistance to shrinkage deformation compared to the 15% NA-C mixture. This is because the primary mechanism by which coarse aggregates mitigate shrinkage is through their high elastic modulus and volumetric restraint, which physically restricts the contraction of the cementitious paste. Coarse aggregates act as a rigid skeleton, absorbing and resisting the tensile stresses generated during drying. The coarse aggregates’ lower specific surface area also means they adsorb less water, but their main contribution is mechanical restraint, not pore size effects [8,16,33]. This indicates that an appropriate amount of NA-C is beneficial for enhancing the mixture’s resistance to drying shrinkage.

### 3.7. Freeze–Thaw Resistance Performance

**Figure 15:**
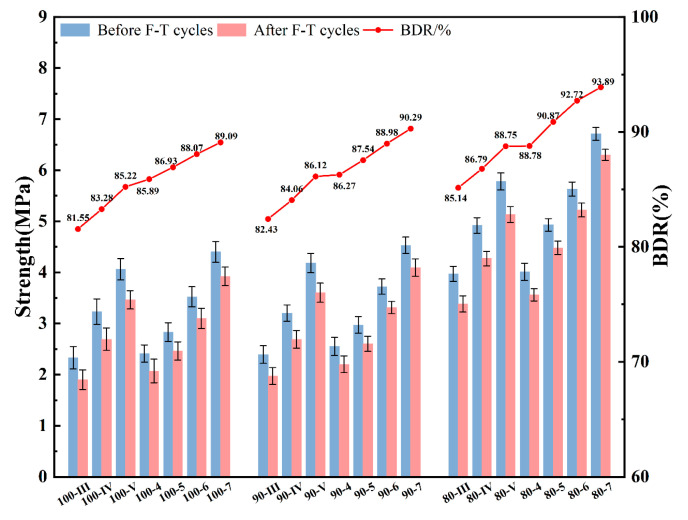
The freeze–thaw cycle test of 28 d.

The freeze–thaw cycle test serves as a critical indicator for verifying the durability of base course mixtures. After undergoing five freeze–thaw cycles, the results are presented in Figure 15. The RBM surface exhibits a rough, porous structure with high water absorption. The 100% RBM mixture contained the highest levels of free water and internal water content. This moisture can permeate and fill the pores within the mixture, adversely affecting the material under low-temperature conditions. As the RBM content decreased, the mixture’s freeze resistance improved, with the most significant enhancement observed in the 80% group. This trend is consistent with findings reported in studies on recycled aggregate mixtures, where higher replacement ratios of recycled materials were found to compromise freeze–thaw durability [17,28].

As evidenced by the experimental data, the 80-7 mixture group attained the highest BDR value of 93.89%, which represents a 4.8% enhancement relative to the 100-7 group. The freeze–thaw resistance of SSRBM specimens compared to their CSRBM counterparts was consistently superior, with frost durability showing marked improvement as RBM content decreases across all mixtures. The underlying damage mechanism is characterized by internal cracking initiated by water phase transformation. When saturated specimens undergo freezing, the volumetric expansion of pore water generates substantial internal stresses, forcing aggregate particles into mutual compression and thereby inducing microcrack formation. With progressive freeze–thaw cycling, this damage accumulates to a critical threshold where microcracks coalesce into interconnected fissures, ultimately leading to significant reduction in compressive strength [34,35]. Consequently, the freeze–thaw deterioration in semi-rigid base materials principally stems from the cyclic expansion of free and capillary water, which induces structural damage through crack propagation and volumetric swelling.

Notably, although CSRBM achieves higher maximum dry density and lower overall porosity than SSRBM, this very density promotes water retention within the matrix. The subsequent expansion of this confined water during freezing creates destructive pressures that disrupt the internal structure, ultimately explaining CSRBM’s compromised frost resistance despite its denser composition. This paradoxical phenomenon has been previously observed in cement-stabilized materials, where denser matrices with higher saturation levels can be more susceptible to freeze–thaw damage due to restricted space for ice expansion [31,34].

## 4. Microstructural Analysis

**Figure 16:**
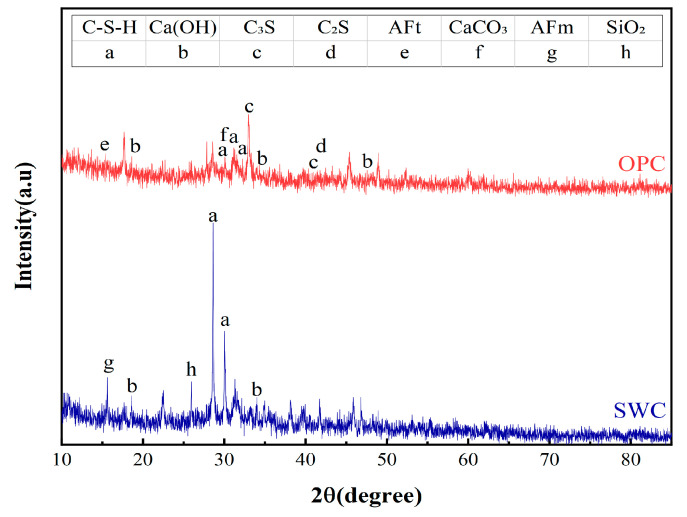
XRD of 28 d OPC and SWC slurry.

Figure 16 displays the X-ray diffraction (XRD) patterns of 28-day ordinary Portland cement (OPC) and slag + waste-derived calcium carbonate (SWC) paste specimens. In the OPC system, diffraction peaks corresponding to C-S-H gel, portlandite (Ca(OH)_2_) and ettringite (AFt)—typical cement hydration products—are identified, together with minor residual peaks of unhydrated alite (C_3_S) and belite (C_2_S). Notably, while no calcium carbonate (calcite) peak is detected in the raw cement powder, a distinct calcite peak emerges in the hydrated paste, indicating that the carbonate phase results from secondary carbonation during or after hydration.

The intensity of the characteristic diffraction peak associated with C-S-H gel is markedly higher in the SWC system than in the pure OPC paste. This enhancement cannot be ascribed to any single highly reactive component; rather, it stems from synergistic interactions within the multi-phase composite system. First, quartz and fine calcium carbonate particles serve as nucleation sites, promoting more ordered precipitation and growth of C-S-H gel. Second, calcium carbonate acts as a reactive filler: it consumes portlandite through the formation of carboaluminate phases and simultaneously stimulates further hydration of silicate constituents, thereby fostering additional C-S-H formation.

**Figure 17:**
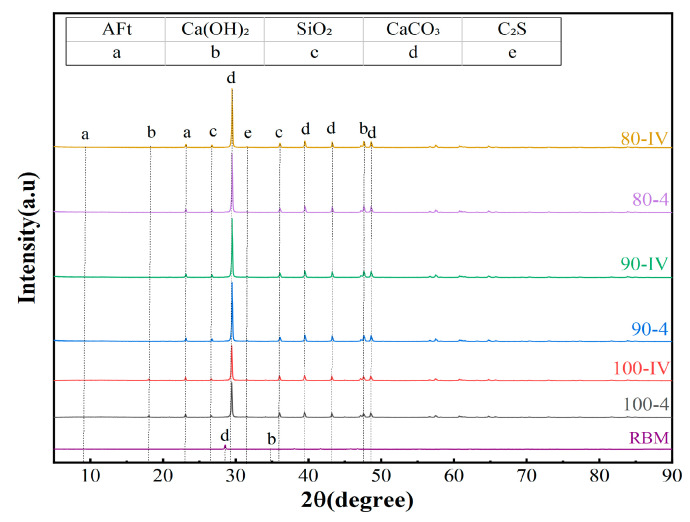
XRD of 90 d CSRBM, 90 d SSRBM, and RBM.

Figure 17 presents the XRD patterns of the 90-day CSRBM, 90-day SSRBM and RBM samples. It can be observed that as RBM content decreases, the phase composition and hydration products in both CSRBM and SSRBM are influenced. The added natural aggregate consists of limestone, and with the extended curing period of 90 days, the intensity of the CaCO_3_ characteristic peak gradually increases in both CSRBM and SSRBM as the proportion of natural aggregate rises.

Comparison between the XRD results of CSRBM and the cement paste indicates that the hydration of cement in the mixture is essentially complete. Moreover, under the same dosage, the characteristic peak intensity of Ca(OH)_2_ in CSRBM remains consistently higher than that in SSRBM, suggesting that the cement-based stabilization of RBM can generate more portlandite.

**Figure 18:**
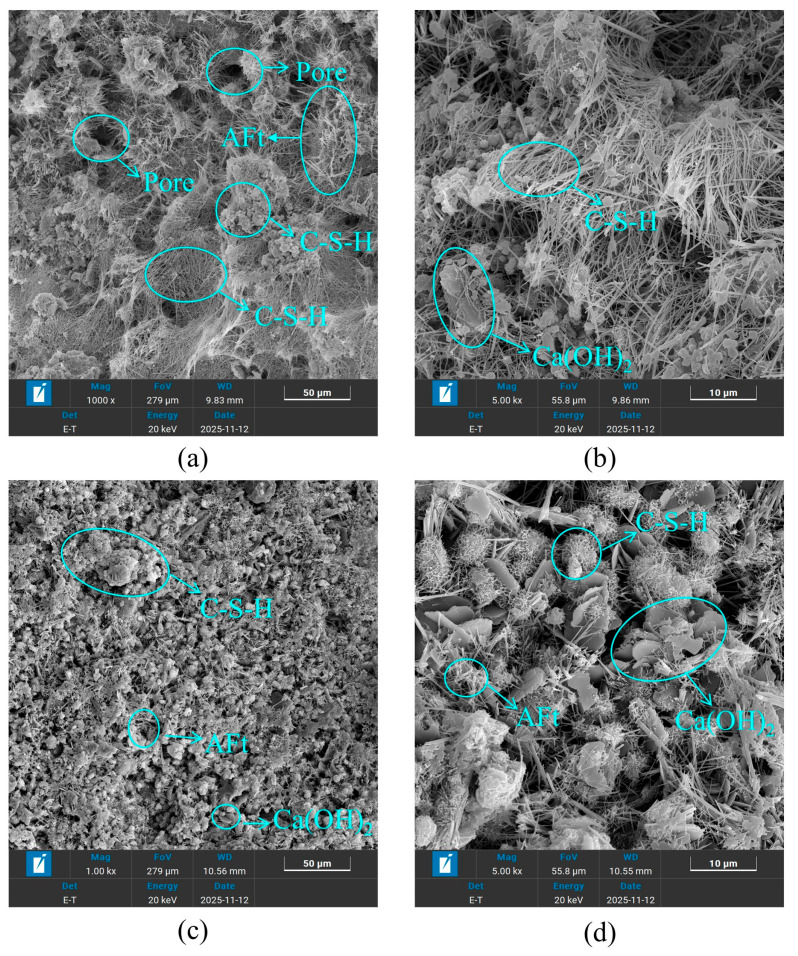
Microstructure of 90 d SSRBM and CSRBM (**a**,**b**); SSRBM and CSRBM (**c**,**d**).

Figure 18a,b present 90-day SEM images of SSRBM (100% RBM, 4% SWC), while Figure 18c,d present 90-day SEM images of CSRBM (100% RBM, 4% OPC). The strength development mechanism of SSRBM is similar to that of CSRBM, primarily relying on the hydration reaction of the cementitious materials. Hydration products (C-S-H gel) form a three-dimensional network structure that effectively bonds RBM and natural aggregate. Ettringite fills the pores, thereby enhancing the micro-scale density of the mixture. Test results indicate that the morphology of hydration products in SSRBM and CSRBM differs. In SSRBM, the C-S-H gel predominantly exhibits a fibrous network structure, whereas in CSRBM, it mainly appears as clustered aggregates. The fibrous C-S-H gel in SSRBM forms a robust three-dimensional network within the material, which facilitates effective stress transfer and dispersion. Simultaneously, it encapsulates larger crushed particles, binding loose granules into an integrated whole. In contrast, the clustered gel structure in CSRBM contributes to a denser internal matrix by effectively filling fine pores, thereby significantly enhancing its strength. When capillary water evaporates from the mixture, causing volumetric shrinkage, the fibrous gel structure in SSRBM establishes a continuous and sturdy three-dimensional framework. This framework effectively resists shrinkage stresses induced by capillary tension. Although the fibrous network may increase porosity, the pores are typically interconnected and tortuous, prolonging the moisture evaporation path. This results in a more gradual drying process, thereby reducing both the shrinkage rate and stress concentration.

Compared to SSRBM, CSRBM generates a substantial amount of Ca(OH)_2_, which has significantly higher solubility than C-S-H gel. This directly introduces unstable and erosion-prone components into the system. Moreover, Ca(OH)_2_ tends to accumulate at the aggregate interface, forming an oriented layer of coarse crystals, which constitutes the weakest zone in the material. Under stress induced by freeze–thaw cycles, microcracks preferentially initiate and propagate within these CH-rich weak areas, directly compromising the material’s ability to resist frost-heaving stresses.

These findings provide a microstructural explanation for the superior resistance to drying shrinkage and freeze–thaw cycles observed in SSRBM compared to CSRBM.

## 5. CO_2_ Emission and Cost Analysis

The CO_2_ emissions of water, aggregates, OPC, and SWC were quantified with reference to Table 10 [27,36,37,38,39]. The material consumption required for producing 1 m^3^ of SSRBM and CSRBM is summarized in Table 11.

The carbon emission data of SSRBM and CSRBM are summarized in Table 12. For CSRBM, cement is the primary source of carbon emissions, accounting for approximately 91% of the total emissions from the mixture. In the SSRBM system, SWC constitutes the major contributor to carbon emissions, representing about 66% of the mixture’s total emissions. The carbon emission of the 100-4 mixture is 19.734 kg CO_2_/m^3^, while that of the 100-IV mixture reaches as high as 72.479 kg CO_2_/m^3^. The carbon emissions of SSRBM are significantly lower than those of CSRBM, with the adoption of SSRBM leading to an approximately 73% reduction in carbon emissions. Therefore, substituting CSRBM with SSRBM can significantly reduce carbon emissions, which holds substantial importance for environmental protection and the green transition of the road industry.

From an economic perspective, the raw material cost of SWC is substantially lower than that of OPC, as it is entirely composed of industrial by-products [6]. Based on current market prices in China, the material cost of SWC is estimated to be approximately 60–70% lower than that of OPC [40]. However, it should be noted that the final cost of SWC-stabilized materials may be influenced by factors such as transportation, pretreatment of industrial by-products, and quality control. A comprehensive life-cycle cost analysis considering production, construction, and long-term maintenance would be valuable for future research to fully assess the economic viability of SWCs [20].

## 6. Conclusions

This study first evaluated the physicochemical properties of solid waste-based cementitious material (SWC), cement, reclaimed base course material (RBM), and natural aggregates. It subsequently investigated the differences in road performance, carbon emissions, and energy consumption between SSRBM and CSRBM under varying RBM contents and stabilizer dosages. Based on this comprehensive evaluation, a comparative summary of the key characteristics, advantages, and limitations of SWC-stabilized materials versus conventional cement-stabilized materials is presented in Table 13. The following conclusions were drawn:

(1) The 7 d UCS of SSRBM with 100% RBM content ranges from 1.88 MPa to 3.78 MPa at 4% to 7% SWC, while the strength range for SSRBM with 90% RBM is 2.05 MPa to 3.98 MPa, and for 80% RBM it is 2.91 MPa to 5.35 MPa. According to strength specifications, mixtures with 100% and 90% RBM content meet the requirements for heavy, medium, and light traffic loads in base courses, while mixtures with 80% RBM content satisfy all grading requirements. This indicates that the gradation of the 80% RBM mixture exhibits the best adaptability.
(2) As the content of SWC and OPC increases, the compressive strength, indirect tensile strength, flexural strength, compressive resilience modulus, and freeze–thaw resistance of the mixtures improve. However, the resistance to drying shrinkage decreases. Specifically, for 100% RBM content, compared to the mixture with 4% SWC, the mixture with 5% SWC showed increases of 14.8% in 90-day compressive strength, 15.4% in indirect tensile strength, 14.6% in flexural strength, and 7.5% in compressive resilience modulus. The 28-day freeze–thaw resistance improved only marginally, while the drying shrinkage resistance decreased by 5.3%.
(3) Adding an appropriate amount of coarse natural aggregate (NA-C) significantly enhances the mechanical strength, compressive resilience modulus, freeze–thaw resistance, and drying shrinkage resistance of both SSRBM and CSRBM. In contrast, the addition of fine natural aggregate (NA-F) shows limited improvement. With 4% SWC content, compared to the mixture with 100% RBM, the mixture with 80% RBM content exhibited increases of 54.9% in 90-day compressive strength, 56.4% in indirect tensile strength, 50.0% in flexural strength, 8.4% in compressive resilience modulus, and 3.3% in 28-day freeze–thaw resistance.
(4) The superior drying shrinkage resistance and freeze–thaw durability of SSRBM compared to CSRBM can be primarily attributed to two factors: the SSRBM system generates a lower content of calcium hydroxide, fundamentally reducing the presence of a soluble and vulnerable phase; meanwhile, the fibrous C-S-H gel network formed in SSRBM effectively resists drying shrinkage stress, and its tortuous pore structure retards moisture migration, thereby enhancing resistance to drying shrinkage.
(5) The carbon emission study indicates that substituting CSRBM with SSRBM delivers significant carbon reduction benefits. In CSRBM, cement contributes to approximately 91% of the carbon emissions, with a unit emission intensity as high as 72.479 kg CO_2_/m^3^. In contrast, the primary emission source in SSRBM is SWC, and its unit emission intensity is only 19.734 kg CO_2_/m^3^, enabling a reduction of approximately 73%. From an economic perspective, SWC is entirely composed of industrial by-products, resulting in a raw material cost approximately 60–70% lower than that of OPC. This substitution strategy, combining significant carbon reduction with clear cost advantages, holds considerable importance for the green transition of the road industry and sustainable infrastructure development.
(6) In summary, this study demonstrates that SWC-stabilized high-content RBM is a technically viable and environmentally advantageous alternative for pavement base construction. The combination of superior mechanical performance, enhanced durability, an approximately 73% carbon reduction, and 60–70% lower raw material costs positions this material as a promising solution for sustainable infrastructure. However, several limitations should be acknowledged. The findings are based on laboratory-scale experiments, and long-term field performance under actual traffic and environmental conditions requires further validation. Additionally, the environmental safety of SWC concerning potential leaching of toxic substances was not assessed and should be addressed in future studies. A comprehensive life-cycle cost analysis considering production, transportation, construction, and maintenance would also be valuable. Future research should focus on full-scale field trials, optimization of SWC composition for different RBM sources, and assessment of long-term durability under various climatic conditions.

## Figures and Tables

**Table 1:** Results of original gradation analysis.

Mesh Size (mm)	Aggregate Percentage (%)		Mean Value (%)
	1	2	
31.5	100.0	100.0	100.0
26.5	97.3	98.3	97.8
19	85.6	83.7	84.7
16	75.6	74.6	75.1
13.2	68.3	64.8	66.6
9.5	56.4	49.7	53.1
4.75	33.5	27.9	30.7
2.36	19.9	16.8	18.4
1.18	12.1	10.6	11.4
0.6	6.1	5.6	5.9
0.3	3.0	2.7	2.9
0.15	1.9	1.6	1.8
0.075	1.2	0.9	1.1

**Table 2:** Coarse aggregate.

Tests	RBM (>4.75 mm)	NA-C (10–20 mm)
Crushing value (%)	19.24	13.52
Needle sheet content (%)	12.48	9.16
Soft stone content (%)	3.12	1.96
Water absorption (%)	9.11	3.23
Apparent density (g/cm^3^)	2.751	2.899

**Table 3:** Fine aggregate.

Tests	RBM (<4.75 mm)	NA-F (0–10 mm)
Plasticity index	11.4	5.6
Organic matter content (%)	0.7	0.5
Sulfate content (%)	0.17	0.12
Water absorption (%)	7.42	2.42
Silt content (%)	3.6	2.0
Apparent density (g/cm^3^)	2.630	2.696

**Table 4:** Primary chemical components.

Composition	MgO	SO_3_	CaO	SiO_2_	Al_2_O_3_	Fe_2_O_3_	K_2_O	Na_2_O
SWC (%)	1.53	5.83	51.87	22.86	8.31	3.12	0.95	0.60
OPC (%)	3.15	3.50	54.78	24.78	7.53	4.00	1.00	0.33

**Table 5:** Performance indicators of SWC and OPC.

Item		SWC	OPC
Standard water requirement (%)		29.4	25.2
Specific surface area (kg·m^−2^)		411	349
Setting time (min)	Initial	162	210
	Final	327	411
Compressive strength (MPa)	3 d	22.1	29.3
	28 d	47.9	55.3
Flexural strength (MPa)	3 d	4.2	5.1
	28 d	5.5	5.8

**Table 6:** Compaction test results.

RBM:NA-F:NA-C	Dosage (%)	Number	OWC (%)	MDD (g·cm^−3^)
100:0:0	4% SWC	100-4	11.5	2.129
	5% SWC	100-5	11.7	2.131
	6% SWC	100-6	11.9	2.135
	7% SWC	100-7	12.3	2.139
	3% OPC	100-III	11.2	2.136
	4% OPC	100-IV	11.4	2.139
	5% OPC	100-V	11.6	2.143
90:10:0	4% SWC	90-4	11.2	2.132
	5% SWC	90-5	11.4	2.136
	6% SWC	90-6	11.6	2.139
	7% SWC	90-7	11.9	2.143
	3% OPC	90-III	10.7	2.139
	4% OPC	90-IV	11.0	2.142
	5% OPC	90-V	11.2	2.145
80:5:15	4% SWC	80-4	10.7	2.135
	5% SWC	80-5	11.0	2.138
	6% SWC	80-6	11.3	2.141
	7% SWC	80-7	11.5	2.145
	3% OPC	80-III	10.3	2.143
	4% OPC	80-IV	10.5	2.146
	5% OPC	80-V	10.8	2.150

**Table 7:** Technical requirements for 7-day UCS.

Layer	Extremely and Extra-Heavy	Heavy Traffic	Medium and Light
Base	4.0–6.0	3.0–5.0	2.0–4.0
Sub-base	2.5–4.5	2.0–4.0	1.0–3.0

**Table 8:** Fitting curve parameters for different ratios (UCS).

Group	a	b	R^2^
100-4	1.28	0.20	0.993
100-5	1.52	0.20	0.953
100-6	2.02	0.18	0.958
100-7	2.75	0.15	0.967
100-III	1.05	0.26	0.949
100-IV	1.52	0.24	0.979
100-V	2.37	0.17	0.977
90-4	1.22	0.24	0.958
90-5	1.65	0.19	0.966
90-6	2.23	0.17	0.944
90-7	2.99	0.14	0.943
90-III	1.14	0.25	0.927
90-IV	1.58	0.23	0.948
90-V	2.50	0.16	0.968
80-4	1.87	0.23	0.999
80-5	2.59	0.19	0.998
80-6	3.18	0.17	0.997
80-7	3.96	0.16	0.999
80-III	1.85	0.23	0.999
80-IV	2.62	0.18	0.995
80-V	3.32	0.17	0.999

**Table 9:** Fitting curve parameters for different ratios (ITS).

Group	a	b	R^2^
100-4	0.11	0.29	0.994
100-5	0.14	0.26	0.986
100-6	0.19	0.22	0.964
100-7	0.20	0.25	0.981
100-III	0.09	0.32	0.965
100-IV	0.15	0.25	0.989
100-V	0.18	0.26	0.968
90-4	0.09	0.34	0.999
90-5	0.15	0.25	0.995
90-6	0.21	0.20	0.988
90-7	0.24	0.21	0.991
90-III	0.11	0.28	0.966
90-IV	0.16	0.25	0.978
90-V	0.25	0.18	0.958
80-4	0.20	0.25	0.998
80-5	0.28	0.20	0.961
80-6	0.28	0.24	0.983
80-7	0.36	0.20	0.970
80-III	0.20	0.24	0.989
80-IV	0.24	0.24	0.985
80-V	0.29	0.25	0.999

**Table 10:** Unit emissions of the raw materials.

Raw Materials	Emission (kg CO_2_/ton)
OPC	800
SWC	160
NA	3.87
RBM	3.24
Water	0

**Table 11:** Material consumption of 1 m^3^ SSRBM and CSRBM.

Components	100-4	100-IV	90-4	90-IV	80-4	80-IV
OPC (ton)	0	0.082	0	0.082	0	0.083
SWC (ton)	0.082	0	0.082	0	0.082	0
NA (ton)	0	0	0.205	0.206	0.411	0.413
RBM (ton)	2.047	2.057	1.845	1.854	1.642	1.651
Water (ton)	0.245	0.244	0.239	0.236	0.228	0.225

**Table 12:** CO_2_ emissions of 1 m^3^ SSRBM and CSRBM.

Components	100-4	100-IV	90-4	90-IV	80-4	80-IV
OPC (kg CO_2_/m^3^)	/	65.815	/	65.908	/	66.031
SWC (kg CO_2_/m^3^)	13.102	/	13.120	/	13.138	/
NA (kg CO_2_/m^3^)	/	/	0.793	0.797	1.589	1.597
RBM (kg CO_2_/m^3^)	6.633	6.664	5.978	6.006	5.321	5.348
Water (kg CO_2_/m^3^)	0	0	0	0	0	0
Total emission (kg CO_2_/m^3^)	19.734	72.479	19.891	72.711	20.048	72.976

**Table 13:** Comparison of SWC-stabilized and cement-stabilized materials.

Aspect	SWC-Stabilized Material (SSRBM)	Cement-Stabilized Material (CSRBM)
Environmental benefit	Low carbon emission (approx. 19.7 kg CO_2_/m^3^, 73% lower than cement); solid waste utilization	High carbon emission (approx. 72.5 kg CO_2_/m^3^); natural resource dependence
Mechanical property	Slightly lower early strength (approx. 80–85% of cement at same dosage); stable later-age growth	High early strength; stable strength development
Durability	Excellent freeze–thaw resistance (BDR 93.9%); good drying shrinkage resistance	Relatively lower freeze–thaw resistance (BDR 89.6%); higher shrinkage cracking risk
Economy	Low raw material cost (industrial by-products); pretreatment cost may apply	High raw material cost; mature production technology
Application characteristic	Higher water demand; approx. 1% higher dosage required to achieve equivalent strength	Moderate water demand; well-established dosage design

## Data Availability

The original contributions presented in this study are included in the article. Further inquiries can be directed to the corresponding author.
